# Impact of interkingdom microbial interactions in the vaginal tract

**DOI:** 10.1371/journal.ppat.1012018

**Published:** 2024-03-08

**Authors:** Shirli Cohen, Kyla S. Ost, Kelly S. Doran

**Affiliations:** University of Colorado Anschutz Medical Campus, Department of Immunology and Microbiology, Aurora, Colorado, United States of America; Duke University School of Medicine, UNITED STATES

## Introduction to the important fungal and bacterial players

Studies from recent years have identified a diverse population of both bacteria and fungi in the human vagina and several community signatures have emerged that are associated with susceptibility to infectious disease. A healthy vaginal bacterial microbiome is most frequently dominated by *Lactobacillus* species [1], yet a combination of other genera are isolated in vaginal swabs in both healthy and diseased states. The vaginal fungal mycobiome is dominated by Ascomycota, of which *Candida* species are the most abundant. Approximately 20% to 30% of healthy human vaginal samples contain detectable *Candida* at a single time point, with nearly all of those being *C*. *albicans*, and 70% of the population is expected to be vaginally colonized with *Candida* longitudinally [2].

**Fig 1:**
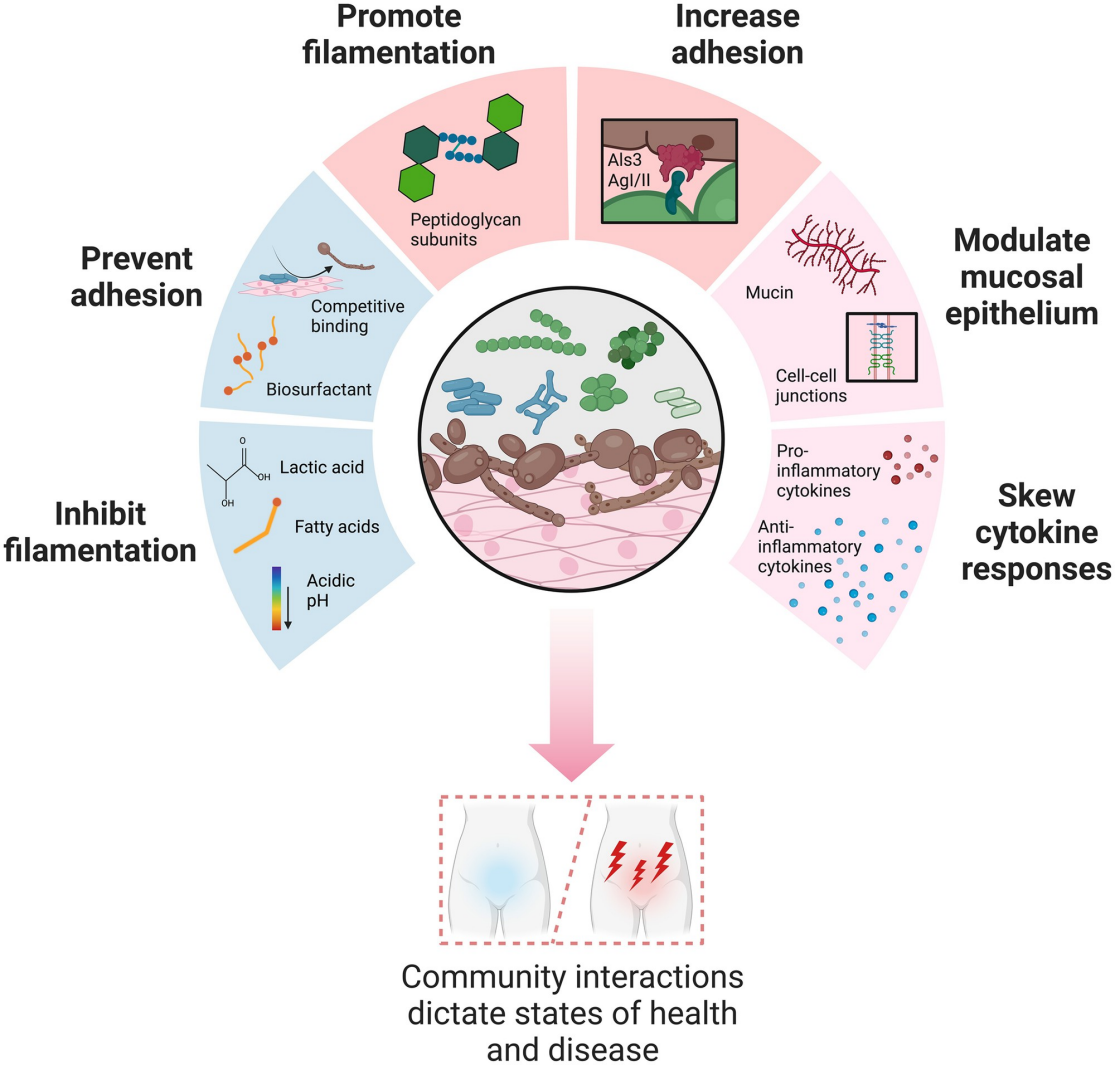
Bacterial interactions with fungi in the vaginal tract take on many forms. Metabolic products, surface proteins, and isolated bacterial components can all impact fungal growth and physiology at the epithelial surface. The vaginal microbiome similarly affects host epithelial biology and immune responses to commensal and pathogenic organisms. In combination, the activities of bacteria and fungi in this environment contribute to the pathology of vaginal infections. Figure created using BioRender.com.

Despite its prevalence in the healthy vaginal mycobiome, *C*. *albicans* is the most common causative agent of vulvovaginal candidiasis (VVC), a cause of significant morbidity in the human population. The next most common species implicated in VVC is *C*. *glabrata*, while other species including *C*. *lusitaniae*, *C*. *tropicalis*, *C*. *parapsilosis*, and *C*. *krusei* are also known to contribute to vaginal infections [3–5]. Non-*albicans Candida* species are typically more resistant to antifungal drugs than *C*. *albicans* [6], complicating therapeutic options and increasing treatment timelines. A disease state is often distinguished by an exaggerated inflammatory response at the vaginal mucosa [3] that leads to vulvar pruritis and erythema [7]. Polymorphic *Candida* species can initiate the process of filamentation, morphogenesis from single-celled yeast to multicellular hyphae, which is typically accompanied by increased tissue damage and inflammation [8]. Microbial dysbiosis has long been associated with VVC due to the observation that antibiotic usage dramatically increases the risk of onset. This notion has been challenged by several population studies [9,10]; however, other studies have found a close association between *Candida* and specific bacteria as well as distinct *Lactobacillus* species that were associated with decreased observation of fungi in samples or symptomatic VVC [11–15]. Taken together, the evidence suggests that specific bacterial species can have complex impacts on fungal burden and symptomatic infection. Shifts in the vaginal microbiome are also associated with other genital tract infections [13,16] including bacterial vaginosis (BV) and aerobic vaginitis (AV), which are characterized by the displacement of *Lactobacillus* species by other bacteria such as *Gardnerella vaginalis*, *Streptococcus agalactiae*, *Staphylococcus aureus*, *Escherichia coli*, and *Enterococcus faecalis* [17]. Both BV and AV are polymicrobial in nature and are associated with concurrent *Candida* carriage in many cases [18,19]. Mixed vaginitis, which can include BV or AV together with VVC, is particularly difficult to treat, and during late stages of pregnancy can lead to severe adverse maternal and neonatal outcomes [17,20]. Because it is the most abundant fungal genus in the vagina, this review will focus on the interactions between *Candida* species and the healthy and pathogenic bacteria found in the vaginal environment. These interactions play a significant role in maintaining homeostasis as well as impacting disease pathogenesis in the genital tract (Fig 1).

## How does the native vaginal microbiome limit fungal infection?

Although the composition of the vaginal microbiome varies between individuals, many are dominated by one species of *Lactobacillus* [21]. This genus is largely considered protective against VVC as lactobacilli secrete numerous factors that can inhibit *Candida* pathogenicity including lactic acid, short and medium chain fatty acids, biosurfactants, bacteriocins, chitinases, and hydrogen peroxide [22]. Lactic acid and fatty acids contribute to the acidic pH typical of the human vaginal tract. Low pH promotes the yeast morphotype [23] of polymorphic *Candida* species which is the less pathogenic morphotype compared to hyphae due to its limited adhesive and invasive capabilities. While this could play a significant role in the vagina, its relevance in humans remains to be determined. Importantly, pH measurements reflect the bulk environment of the vaginal lumen. In its local environment, *C*. *albicans* can modify the pH [24], metabolize lactic acid [25], and respond to extracellular lactate by remodeling its cell wall to shield immune stimulatory glycans [26]. *Lactobacillus*-derived biosurfactants [27] have been reported to interrupt fungal adherence to epithelial cells, limiting opportunities for invasion and tissue damage. Bacteriocins and chitinases produced by lactobacilli directly attack *Candida* species [28], hydrolyzing the fungal cell wall and forming pores. Broadly, the secretome has been shown to reduce *Candida* adherence and biofilm-associated gene expression [29–31]. Beyond secreted factors, *Lactobacillus* species also associate closely with vaginal epithelial cells which reduces *Candida* adherence by way of competitive binding.

The relevance of hydrogen peroxide production on restricting the growth of fungal pathogens in vivo is debated in the literature [32] due to the requirement for oxygen in its biosynthesis and the high concentrations necessary to prevent hyphal morphogenesis. Physiological concentrations of hydrogen peroxide in the vagina are 5 to 25 μm, and *C*. *albicans* growth restriction begins in the mM range; however, local concentrations likely exceed the measured quantities and may be sufficient for inhibition. Furthermore, *L*. *crispatus*, which produces peroxide, is most associated with protection against *C*. *albicans* vaginal infection while *L*. *iners*, which does not produce peroxide, is regarded as least protective and often co-occurs with pathogenic microbes. An *L*. *iners*-dominated vaginal microbiome is thus associated with a higher risk of VVC [13,14,33].

*Bifidobacterium* species are also documented commensals of the vaginal microbiome, yet their interactions with fungal species in the genital tract are less well studied. Human gastrointestinal *Bifidobacterium* isolates have been shown to inhibit *C*. *albicans* growth [34]. Other studies have demonstrated that they can attenuate the pathogenesis of *C*. *albicans* and *Clostridioides difficile* gut coinfections [35]. These results may indicate a protective role for *Bifidobacterium* in the vagina, yet VVC diagnosis, particularly *C*. *glabrata* VVC, has been associated with an increase in relative *Bifidobacterium* abundance [36]. Thus, further studies are required to dissect the role of bifidobacteria in *Candida* pathogenesis.

## How do pathogenic bacteria interact with *Candida* during infection?

Interactions between *C*. *albicans* and the pathogenic bacteria that it encounters have been described for several host environments including the oral cavity, skin, lungs, gastrointestinal tract, and blood. In many cases, fungal–bacterial interactions promote disease severity and increase antimicrobial resistance. Similarly to these other niches, *Candida* species often co-occur with bacterial opportunistic pathogens in the genital tract.

The bacteria that *C*. *albicans* interacts with are known to modulate its adhesive abilities and biofilm formation. *C*. *albicans* encodes a repertoire of adhesive surface proteins that are critical for its adhesion to a wide range of substrates. Additionally, these adhesins mediate physical interactions with other microbes including *S*. *aureus*, a causative agent of AV that has been co-isolated with *C*. *albicans* in vaginal swabs. *S*. *aureus* has been described in association with *C*. *albicans* in numerous studies [20,37,38]. They are known to form robust polymicrobial biofilms together, a physical interaction mediated in part by the agglutinin-like sequence (Als) family of adhesins. *S*. *aureus* does not inhibit hyphal morphogenesis in co-culture with *C*. *albicans*; however, studies have shown that its purified alpha-hemolysin can inhibit filamentation and is protective in an in vivo model of VVC [39]. Alternatively, peptidoglycan subunits from both *S*. *aureus* and *E*. *coli* promote *C*. *albicans* filamentation [40,41]. *E*. *coli*, which has also been co-isolated with *C*. *albicans* in vaginal swabs [42], dramatically alters the structure of a *C*. *albicans* biofilm, presumably by affecting fungal factors such as filamentation that are involved in biofilm formation and maintenance. Work in vitro demonstrates that *Candida* and *E*. *faecalis*, a pathobiont found in the vaginal tract [43,44], communicate using secreted factors controlled by the Fsr quorum-sensing system including EntV, which reduces *C*. *albicans* adhesion and filamentation [45,46]. Quorum sensing is also well known to play a role in *Candida* interactions with *S*. *aureus*. *C*. *albicans* induces the accessory gene regulator (agr) system in *S*. *aureus* [47], and the fungal quorum-sensing molecule farnesol up-regulates staphylococcal efflux pumps [48]. It is unknown whether this occurs in the genital tract and what the implications are for *C*. *albicans* carriage, but this warrants further studies on the consequences of their co-occurrence in this environment.

*S*. *agalactiae*, also known as Group B *Streptococcus* (GBS), is a pathobiont that colonizes the vaginal tract and is associated with pregnancy complications including chorioamnionitis, preterm premature rupture of membranes, and preterm labor, as well as invasive infections in the newborn [49,50]. Many population studies show a significant association between GBS and *C*. *albicans* vaginal carriage [51–55]. *C*. *albicans* has been shown to increase GBS adherence to vaginal epithelial cells due to co-association mediated by the fungal agglutinin-like sequence protein 3 (Als3) and GBS adhesins in the antigen I/II (AgI/II) protein family [56]. The contribution of Als3 to polymicrobial interactions is further illustrated by GBS preferentially binding to hyphae, which express Als3 on their surface, compared to the yeast morphotype of *C*. *albicans*, which do not. There is also evidence that GBS impacts *C*. *albicans* pathogenesis. In vitro, GBS may inhibit *C*. *albicans* filamentation as well as increase fungal burdens in a murine model of vaginal infection [57]. A growing body of work describes the mechanisms of *C*. *albicans* interactions with other streptococci in the oral cavity [37,58,59]. This raises the question of whether *C*. *albicans* and GBS are also interacting through extracellular signals and secreted metabolites in addition to adhesin-mediated physical binding.

Population studies of the human vaginal microbiome have revealed associations between BV pathogens and fungi. *Gardnerella vaginalis* is positively correlated with *C*. *lusitaniae* VVC and has been imaged in direct association with invasive *Candida* hyphae in human biopsy samples [11]. *Prevotella bivia* is highly abundant in individuals with *C*. *glabrata* VVC, and *Peptostreptococcus* species are positively correlated with *C*. *parapsilosis* VVC. These patterns suggest that individual species of *Candida* may have unique bacterial interaction profiles.

## What aspects of the host environment dictate interactions?

In addition to directly antagonizing *C*. *albicans* pathogenicity, *Lactobacillus* species may support host defenses against VVC. They have been shown to increase mucus production in the vagina as well as promote tight junction maintenance and antimicrobial peptide production [28,60]. These factors likely play a role in preventing fungal invasion into the epithelium and subsequent tissue damage. Mucosal epithelial features of the vaginal environment greatly influence *C*. *albicans* pathogenicity and can be modulated by resident microbes. *C*. *albicans* secreted proteases hydrolyze hemoglobin present in the lumen, producing antimicrobial hemocidins [61] with broad activity against *L*. *acidophilus* and *E*. *coli*. Additionally, *C*. *albicans* can initiate the release of arachidonic acid from host cells [62], which it can not only utilize as a carbon source to promote growth but also to generate oxylipins [63]. These have been shown to contribute to weakened tight junctions and an increased inflammatory response.

Mounting evidence strongly indicates that VVC symptoms are mediated by the host inflammatory response to fungal factors [3,64]. Interestingly, a vaccine that enhances an Als3-specific antibody response is protective against both recurrent VVC as well as *S*. *aureus* infection [65–67]. This suggests that a shared epitope may mediate cross-kingdom protection or that an immune response against *Candida* could promote *S*. *aureus* clearance indirectly. Further, this highlights that in the absence of vaccination, the inflammatory response is nonprotective against these 2 pathogens and may cause immunopathology during infection. *L*. *crispatus* could play a role in dampening the native nonprotective response against *C*. *albicans* by altering the cytokine profile [68]. Among these alterations, it has been demonstrated to induce IL-1RA and IL-2 secretion in vaginal epithelial cells and inhibit IL-6, IL-8, TNFα, RANTES, and MIP3α during in vitro infection with *C*. *albicans*. IL-1RA has a demonstrated role in protection against pathogenic inflammation during VVC [69,70], and these findings highlight an avenue for further investigation into the activity of IL-2 and other cytokines in this environment. Together, this may support a potential role for select lactobacilli in suppressing inflammatory immune responses and preventing VVC pathogenesis. *L*. *crispatu*s has also been shown to increase IL-17 release by vaginal epithelial cells in response to *C*. *albicans* infection [60], yet it is unclear whether this is protective against VVC. Some studies show that a Th17 response is beneficial while others dispute this, indicating that it plays a nuanced role in this environment [71–74]. Recruited neutrophils can exacerbate VVC pathology [8,75–77], yet antimicrobial peptide production involved in the Th17 response can facilitate fungal clearance [71]. Although mechanistic studies in humans are limited, human genetic deficiencies in IL-17 and IL-22 production have been associated with a higher risk of VVC [78,79]. Interestingly, GBS attenuates the Th17 immune response to *C*. *albicans* in a murine model of VVC [57], which may contribute to increased fungal burdens in the vaginal lumen during coinfection. The role of several cytokine responses to VVC has been reviewed in depth [72,80].

## Conclusion

The diverse communities of bacteria and fungi in the genital tract lend themselves to an equally diverse array of interkingdom interactions. Understanding the mechanisms by which these interactions occur and impact vaginal health is critical to developing therapeutic strategies to treat infection in this dynamic and polymicrobial environment. Significant work remains to characterize the interkingdom interactions that occur in this unique host niche.
